# Social and Poor-Law Problems

**Published:** 1908-06-13

**Authors:** 


### SOCIAL AND POOR-LAW PROBLEMS.

### DR. MACNAMARA AND THE POOR-LAW CHILDREN.

Under the auspices of the Superintendents of Poor-law Schools Association, a number of the gentlemen connected with it went recently to the office of the Metropolitan Asylums Board on Victoria Embankment to hear an address from Dr. Macnamara on "The Care of the Child under the Poor Law." Dr. Macnamara said some kind things of the present system of dealing with Poor-law children, but he did not use the language of flattery. Among the satisfactory things he noted that of the 69,000 children now chargeable to the Poor Law, only 565 are actually living and being educated in workhouses, while about half of them attend the public elementary schools. There are 8,700 being cared for in infirmaries, the infirm wards of workhouses, and in the many institutions belonging to the Metropolitan Asylums Board. These include schools to which children are transfered when suffering from such diseases as ringworm, which do not preclude the children from going on with their education. About 14,000 still remain in workhouses—but many of these are infants under school age, 12,000 are in district or separate schools, 11,000 in cottage homes, 5,000 in scattered homes, 9,300 in institutions for the mentally and physically defective, and 8,700 are boarded out. Dr. Macnamara offered no opinions as to which of these places are the best for the Poor-law child, but speaking as an old teacher he reminded his hearers that those who have the care of Poor-law children have to fill the part not only of teachers but of parents, and that to a class which starts as a rule heavily handicapped physically, morally, and intellectually. Dr. Macnamara seemed to think that the physical handicap is ultimately overcome, for the children in Poor-law schools are better fed and clothed than those in the poorer schools outside, but intellectually the Poor-law child remains below the average. He is delighted with the good behaviour and kindly spirit of Poor-law children, but finds them deficient in intellectual grip and concentration. He suggested that teaching service in Poor-law schools should be made interchangeable with teaching in outside elementary schools. This seems a very good suggestion, for it must be deadening to the teacher's faculties to be confined all his or her life to working among these handicapped children, while a teacher coming from the outside world, fresh from contact with the more vigorous, even if occasionally more troublesome, child of the ordinary school, would bring in new ideas and perhaps new methods which might awaken dormant powers in his Poor-law pupils. In conclusion Dr. Macnamara reminded his hearers that the true test of the success of their work lay in the after careers of their pupils, and he urged guardians to keep in touch with the various institutions which befriend boys and girls after they leave school.

